# Species-specific effects of the introduction of *Aspergillus nidulans gfdB* in osmophilic aspergilli

**DOI:** 10.1007/s00253-023-12384-9

**Published:** 2023-02-22

**Authors:** Veronika Bodnár, Anita Király, Erzsébet Orosz, Márton Miskei, Tamás Emri, Zsolt Karányi, Éva Leiter, Ronald P. de Vries, István Pócsi

**Affiliations:** 1 Department of Molecular Biotechnology and Microbiology, Institute of Biotechnology, Faculty of Science and Technology, University of Debrecen, Debrecen, Hungary; 2 Doctoral School of Nutrition and Food Sciences, University of Debrecen, Debrecen, Hungary; 3 ELRN-UD Fungal Stress Biology Research Group, Debrecen, Hungary; 4 Department of Medicine, Faculty of Medicine, University of Debrecen, Debrecen, Hungary; 5 Fungal Physiology, Westerdijk Fungal Biodiversity Institute & Fungal Molecular Physiology, Utrecht University, Utrecht, the Netherlands

**Keywords:** *Aspergillus nidulans*, *Aspergillus wentii*, *Aspergillus glaucus*, Osmophily, Environmental stress, Oxidative stress tolerance

## Abstract

**Abstract:**

Industrial fungi need a strong environmental stress tolerance to ensure acceptable efficiency and yields. Previous studies shed light on the important role that *Aspergillus nidulans gfdB*, putatively encoding a NAD^+^-dependent glycerol-3-phosphate dehydrogenase, plays in the oxidative and cell wall integrity stress tolerance of this filamentous fungus model organism. The insertion of *A. nidulans gfdB* into the genome of *Aspergillus glaucus* strengthened the environmental stress tolerance of this xerophilic/osmophilic fungus, which may facilitate the involvement of this fungus in various industrial and environmental biotechnological processes. On the other hand, the transfer of *A. nidulans gfdB* to *Aspergillus wentii*, another promising industrial xerophilic/osmophilic fungus, resulted only in minor and sporadic improvement in environmental stress tolerance and meanwhile partially reversed osmophily. Because *A. glaucus* and *A. wentii* are phylogenetically closely related species and both fungi lack a *gfdB* ortholog, these results warn us that any disturbance of the stress response system of the aspergilli may elicit rather complex and even unforeseeable, species-specific physiological changes. This should be taken into consideration in any future targeted industrial strain development projects aiming at the fortification of the general stress tolerance of these fungi.

**Key points:**

*• A. wentii c’ gfdB strains showed minor and sporadic stress tolerance phenotypes.*

*• The osmophily of A. wentii significantly decreased in the c’ gfdB strains.*

*• Insertion of gfdB caused species-specific phenotypes in A. wentii and A. glaucus.*

**Supplementary Information:**

The online version contains supplementary material available at 10.1007/s00253-023-12384-9.

## Introduction

Many industrial microbial biotechnological processes take advantage of the remarkable diversity, productivity, technological applicability, and high endurance of the ascomycete fungi belonging to the genus *Aspergillus* (de Vries et al. 2017; Park et al. 2017; Cairns et al. 2021; Huang et al. 2021; Jin et al. 2022). For example, the xerophilic/osmophilic *Aspergillus wentii* (Wheeler and Hocking 1993; de Lima Alves et al. 2015; de Vries et al. 2017) is a good enzyme producer (Sinha and Chakrabarty 1978; Chander et al. 1980; Gross et al. 1984; Lago et al. 2021) and can be a workhorse in future biodiesel transesterification processes as well (Shoaib et al. 2018).

Based on previous hyperosmotic stress studies, osmophily is widespread in the aspergilli and the growth stimulatory effect of either 2.0 M sorbitol or 1.0 M NaCl was the most significant for *A. wentii* and *Aspergillus glaucus* (de Vries et al. 2017). In these fungi, osmophily was hypothesized to be connected to the lack of the *gfdB* gene, encoding a putative NAD^+^-dependent glycerol-3-phosphate dehydrogenase enzyme in many *Aspergillus* spp., including the filamentous fungus model organism *Aspergillus nidulans* (Miskei et al. 2009; Balázs et al. 2010; de Vries et al. 2017). Importantly, the xerophilic/osmophilic *A. glaucus* may also find its industrial fermentation applications in enzyme (Tao et al., 2010, 2011; Abrashev et al. 2016; Li et al. 2018; Takenaka et al. 2019) and aspergiolide A (an anticancer-polyketide) (Cai et al. 2009, 2014; Sun et al. 2009; Wu et al. 2017) production, and *A. nidulans* is also a well-known enzyme producer fungus and represents a potential platform for heterologous protein expression (MacCabe et al. 2002; Kumar 2020; Lopes et al. 2021; Jin et al. 2022). It is noteworthy that *A. glaucus*, which has a remarkably high abiotic stress resistance, is also considered a suitable tool in saline-alkaline remediation technologies (Wei and Zhang 2018; Zhou et al. 2021), in the biomineralization of the insecticide fipronil (Gajendiran and Abraham 2017), and in the hydrolysis of sugar cane bagasse (Tao et al. 2010). *A. glaucus* stress tolerance genes transferred into other organisms can enhance the osmotic stress tolerance of recipient fungi and plants (Liu et al. 2014, 2015).

Unexpectedly, the deletion of *gfdB* resulted in a decreased oxidative and cell wall integrity stress tolerance in *A. nidulans* (Király et al. 2020a) although its expression was only responsive to 0.6 M NaCl exposure and not to oxidative stress (Balázs et al. 2010). Nevertheless, the hyphal-morphology of the *A. nidulans ΔgfdA* mutant strain was altered, and its growth was also attenuated at various carbon sources except glycerol (Fillinger et al. 2001). This defective growth was recoverable on all carbon sources in the presence of 1 M NaCl, which functioned as an osmotic stabilizer for the mutant strain. Furthermore, the mutant strain was also sensitive to cell wall integrity stress (Fillinger et al. 2001).

In *A. glaucus*, the implementation and expression of *A. nidulans gfdB* with its own promoter increased considerably the oxidative, cell wall integrity, and heavy metal (Cd^2+^) stress tolerance of the fungus cultured on 2 M sorbitol without affecting its osmophily (Király et al. 2020b). Because the adequate stress tolerance of industrial yeasts and filamentous fungi is of paramount importance in fermentation processes (Bai et al. 2003; Li et al. 2011; Teixeira et al. 2011; Hagiwara et al. 2016; Deparis et al. 2017; Steensels et al. 2019; Brandt et al. 2021; Yaakoub et al. 2022), we wanted to test if the genetic transfer of *A. nidulans gfdB* to *A. wentii* with its own promoter would also enhace the general stress tolerance of this fungus.

The outcomes of this study supported the original hypothesis of Miskei et al. (2009) on the possible involvement of GfdB in the control of xerophilic/osmophilic phenotypes, and not in the environmental stress tolerance of the aspergilli. The significance of the appearant species-specific physiological functions of GfdB has also been discussed here together with the implications for the future development of stress-tolerant industrial fungal strains.

## Materials and methods

### Fungal strains and culture conditions

The list of strains used in this study is presented in Table 1. *A. wentii* and *A. glaucus* conidia were produced on Malt Extract Agar (MEA) (1.5 % agar, 25 °C in the dark, 6 days), and all sporulation media were supplemented with 1.0 M NaCl in the case of *A. glaucus* (de Vries et al. 2017; Emri et al. 2018; Orosz et al. 2018). *A. nidulans* strains were sporulated on Barratt's nitrate minimal medium (NMM) under standard conditions (1.5 % agar, 25 °C in the dark, 6 days; (Barratt et al. 1965). Conidiospores were scraped in sterile water containing 9 g L^-1^ NaCl and 100 μL L^-1^ TWEEN-80, passed through two layers of Miracloth (Merck-Millipore, Burlington, MA, USA) and then quantified using a hemocytometer. All strains were grown on NMM nutrient agar plates under the culture conditions indicated and were stored in conidiospore suspension stocks prepared in 50 % glycerol and were kept at -75 ^o^C (Szabó et al. 2020a,b).

**Table 1:** *Aspergillus* strains used in this study

Strain name	Relevant genotype	References
*A. nidulans* THS30.3^a^	*pyrG89*, *AfupyrG*^*+*^; *pyroA*^*+*^; *veA*^*+*^; prototrophic control	(Park et al. 2015)
*A. nidulans ΔgfdB*^a^	*pyrG89*; *ΔgfdB*::*AfupyrG*^+^; *pyroA*^+^; *veA*^+^	(Király et al. 2020a)
*A. glaucus* CBS516.65	Wild-type	(de Vries et al. 2017)
*A. glaucus* CBS516.65 *'c gfdB1*, *'c gfdB2*	*gfdB* supplemented	(Király et al. 2020b)
*A. wentii* CBS141173	Wild-type	(de Vries et al. 2017)
*A. wentii* CBS141173 *'c gfdB1, 'c gfdB2*, *'c gfdB3*	*gfdB* supplemented	This study

^a^*A. nidulans* strains are maintained in the strain collection of the Department of Molecular Biotechnology and Microbiology, University of Debrecen

### Generation of the *A. nidulans gfdB* complemented strains of *A. wentii*

The plasmid pAN7.1 (Punt et al. 1987; full sequence map is available at https://www.addgene.org/168129/) containing the hygromycin B resistance gene was used to transform *A. wentii* protoplasts with the *A. nidulans gfdB* (locus ID: AN6792) gene with its native promoter and terminator sequences (for primer pair see Supplemental Table S1), which was cloned to the *Xba*I-*Hin*dIII site. Protoplasts were generated from exponential growth phase (13-14 h old) submerged cultures of *A. wentii* grown on complex medium (NMM containing 2% glucose and supplemented with 0.5% yeast extract and 1% peptone) using the lysing enzymes from *Trichoderma harzianum* (Sigma, St Louis, MO, USA) with the polyethylene glycol (PEG)-mediated transformation method as previously described in the protocol of Szewczyk et al. (2006). We used 10^6^–10^7^ protoplasts in 100 μl suspension and 5–8 μg pAN7.1 plasmid in 10 μl aliquot per transformation. Transformants were regrown from a single conidium on NMM containing 100 μg mL^−1^ hygromycin at 25 °C after 3–5 days incubation. For the genomic DNA isolation, transformants were incubated in a rotary shaker overnight at 25 °C, 220 rpm in NMM containing 100 μg mL^−1^ hygromycin. Genomic DNA was isolated from mycelial mat collected by centrifugation (Szabó et al. 2020a). To prove the successful incorporation of the *gfdB* gene, after genomic DNA isolation, Emerald PCR reactions (EmeraldAmp MAX PCR Master Mix, Takara Bio, San Jose, CA, USA) were carried out with the *AN6792 Xba*I FW and *AN6792 Hin*dIII REV primers (Supplemental Table S1).

### Copy number analysis of the *gfdB* gene using the quantitative polymerase chain reaction (qPCR) method

For the qPCR reaction, we used the Fast SYBR® Green master mix (Applied Biosystems by Life Technologies, Waltham, MA, USA) kit. To determine the copy number of the *gfdB* gene, a dilution series (320 ng, 160 ng, 80 ng, 40 ng, 20 ng, 10 ng DNA per 7 μl volume) was prepared from the genomic DNA of *A. wentii* transgenic strains of known concentration. The single copy *Aspwe1*_39921 gene, encoding the *A. nidulans* γ-glutamylcysteine synthetase (locus ID: AN3150) ortholog in *A. wentii*, was used as a copy reference gene. qPCR reactions were carried out in 96 well plates, and the reaction mixtures contained 7 μl of a given concentration of genomic DNA, 10 μl of Fast SYBR® Green master mix, 0.4 μl of forward primer, 0.4 μl of reverse primer, and 2.2 μl of nuclease-free water. Three parallel measurements were performed with each primer pair at each genomic DNA concentration (for the complete list of primer pairs see Supplemental Table S1) on a LightCycler®480 equipment (Roche, Basel, Switzerland). PCR cycles were performed according to the following protocol: 1. 95 °C 2 min; 40× cycles: 95 °C 5 s, 51 °C 10 s, 65 °C 20 s; 95 °C 15 s, 51 °C 15 s, 95 °C continuous, 37 °C 1 s (Szabó et al. 2020a).

The copy number of the *gfdB* gene incorporated in the *A. wentii* genome was quantified as previously described {(Király et al. 2020a, b; Szabó et al. 2020a, b); using the equation of (Herrera et al. 2009)}.

$$gfdB\ \textrm{copies}\ \textrm{per}\ \textrm{genome}=\left(\textrm{total}\ \textrm{copies}\ \textrm{of}\ \textrm{gfdB}\right)/\left(\textrm{total}\ \textrm{copies}\ \textrm{of}\ \textrm{Aspwe}\_39921\right)$$

### Determination of *gfdB* gene expression by quantitative real-time reverse transcription PCR (qRT-PCR) in *A. wentii 'c gfdB* complemented strains

For the RNA isolation, mycelial samples were collected after 3-day incubation in a rotary shaker (NMM, 220 rpm, 25 °C). Total RNA was isolated from lyophilized mycelia using TRIzol reagent (Chomczynski 1993), and qRT-PCR experiments were performed as described earlier (Emri et al. 2015) using a Xceed SG 1-step 2× Mix Lo-ROX qPCR Kit (Institute of Applied Biotechnologies, Prague, Czech Republic). In qRT-PCR measurements, 500 ng of total RNA per reaction was added and the reactions were halted after 40 cycles according to the manufacturer’s recommendations. The applied primer pairs are summarized in Supplemental Table S1. Relative transcription levels were quantified with the ΔΔCP method, where ΔC_T_ = C_T_ reference gene − C_T_ *gfdB*, and C_T_ stands for the qRT-PCR cycle numbers corresponding to the crossing points. Relative transcript levels were also examined using the following reference gene: *Aspwe1_38228* (*A. fumigatus tef1* ortholog), and these reactions gave similar results (Szabó et al. 2020a).

### Stress tolerance studies

Large-scale stress agar plate assays were performed (Balázs et al. 2010) to study and compare the stress sensitivities of the tested reference and mutant strains (Table 1**)**. Following standard stress agar plate protocols routinely used in our laboratory (de Vries et al. 2017, Orosz et al. 2018), 1×10^5^ freshly harvested spores were point-inoculated on Barratt’s NMM agar and were incubated at 25 °C for 5 and 10 days. NMM agar was supplemented with various stress-generating agents as required. For the osmophilic *A. wentii* and *A. glaucus* strains, a similar set of stress sensitivity experiments was repeated where NMM agar was also supplemented with 2 M sorbitol in addition to the stressors. The following stress-eliciting agents were employed at the concentrations indicated in parentheses: cell wall integrity stress: Congo Red (54, 81, and 108 μM); oxidative stress: *tert*-butyl hydroperoxide (*t*BOOH; 0.4, 1.6, and 2.4 mM), hydrogen peroxide (9 and 18 mM), menadione sodium bisulphite (MSB; 0.096, 0.19, 0.38, 0.62 mM), diamide (1.5 mM); heavy metal stress: CdCl_2_ (0.1, 0.15, 0.2, and 0.5 mM); hyperosmotic stress (when this tearm is applicable)): sorbitol (2 M), NaCl (0.5, 1, and 1.5 mM). Following stress treatments, the stress sensitivity of the strains was characterized by the diameters of the colonies (Balázs et al. 2010; de Vries et al. 2017; Orosz et al. 2018; Király et al. 2020a,b).

### Cluster analysis and multidimensional scaling of stress tolerance

To perform cluter analysis on the general stress sensitivity observed in the genus of the aspergilli, growths of the *A. wentii* (CBS141173), *A. wentii* (CBS141173) *'c gfdB1*, *A. glaucus* (CBS516.65), *A. glaucus* (CBS516.65) *'c gfdB1*, *A. nidulans* THS30.3, and *A. nidulans ΔgfdB* strains recorded in current and pervious studies (Király et al. 2020a,b) were compared to growth data gained with other *Aspergillus* spp. and deposited in the Fungal Stress Database (FSD; https://www.fung-stress.org/; de Vries et al. 2017; Orosz et al. 2018; Emri et al. 2018). Other *Aspergillus* species (15 spp. in total) whose stress sensitivity data are available in the Fungal Stress Database include the following species: *A. aculeatus* (CBS 172.66), *A. brasiliensis* (CBS 101740), *A. carbonarius* (CBS 141172 = DTO 115-B6), *A. clavatus* (CBS 513.65 = NRRL1), *A. fischeri* (CBS 544.65), *A. flavus* (CBS 128202 = NRRL 3357), *A. fumigatus* (CBS 126847 = Af293), *A. luchuensis* (CBS 106.47), *A. niger* (represented by two strains: CBS 113.46 and N402), *A. oryzae* (Rib40), *A. sydowii* (CBS 593.65), *A. terreus* (NIH2624), *A. tubingensis* (CBS 134.48), and *A. versicolor* (CBS 795.97).

As described before (Emri et al. 2018), MIC_50_ values, which were defined as the lowest concentrations of the tested stress initiating agents, causing 50% decreases in colony growth, were calculated for stress agar cultures exposed to H_2_O_2_, MSB, and CdCl_2_ at 25 °C for 5 and 10 days. In the case of NaCl, Congo Red and sorbitol, relative growth values (% of those recorded in unstressed control cultures) measured at 1.0 M, 108 μM, and 2.0 M concentrations, respectively, were taken into consideration on the cluster analyses, which were performed with the R version 4.2.0 software. The values were standardized for comparability, and the data are available in Supplemental Table S2. The “dist,” “hclust,” and “cmdscale” functions of the R Project (www.R-project.org/) were used to calculate Euclidian distances between strains and to generate the cladogram and MDS plot, respectively.

Similarities and differences between the stress tolerance of the *A. wentii* CBS141173, *A. wentii* CBS141173 *'c gfdB1*, *'c gfdB2* and *'c gfdB3*, *A. glaucus* CBS516.65, *A. glaucus* CBS516.65 *'c gfdB1* and *'c gfdB2* as well as *A. nidulans* THS30.3 and *A. nidulans ΔgfdB* strains based on the mean colony diameter values recorded under different stress conditions were also visualized with MDS. In this case, colony diameters, measured in the presence of the following stressors: 54 μM Congo Red, 2 M sorbitol, 0.096 mM MSB, 0.4 mM *t*BOOH, 9 mM H_2_O_2_, 1 M NaCl, 0.5 mM CdCl_2_, were taken into consideration to compare the strains. In the case of the highly osmophilic *A. glaucus*, all NMM stress agar plates were supplemented with 2 M sorbitol.

### Statistical analysis

The effects of stress treatments and gene manipulations on the growth of the *A. wentii* CBS141173, *A. wentii* CBS141173 *'c gfdB1*, *'c gfdB2* and *'c gfdB3*, as well as the *A. glaucus* CBS516.65 and *A. glaucus* CBS516.65 *'c gfdB1* and *'c gfdB2* strains were analyzed by two-way ANOVA followed by Tukey’s post hoc test. The difference between the mean colony diameter values were considered significant if the adjusted *p*-value was less than 0.05 (Király et al. 2020a,b).

## Results

### Supplementation of *A. wentii* with *A. nidulans gfdB* gene and phenotypic characterization of the *A. wentii* wild-type and ’*c gfdB* strains

To construct *A. wentii ’c gfdB* mutant strains, the pAN7.1 plasmid, containing the *A. nidulans gfdB* gene with its native promoter and terminator sequences, was introduced in *A. wentii*. The presence of the *gfdB* gene was verified with the *AN6792 Xba*I FW and *AN6792 Hin*dIII REV primers (Supplemental Table S1). After the verification of the expected genotypes, the copy number of the incorporated *A. nidulans gfdB* gene (Table 2) was determined, and the expression of *gfdB* in *A. wentii* was demonstrated by qRT-PCR method (Supplemental Fig. S1).

**Table 2:** Determination *gfdB* copy number in *gfdB* supplemented *Aspergillus wentii ’c gfdB* strains^a^

Strains	*gfdB*^b^	*r*^2^	*Aspwe1_39921*^b^	*r*^2^	Copy number
*’c gfdB1*	y=−4.2037×+23.034	0.98	y = −4.5586× + 28.023	0.99	0.91 ± 0.18
*’c gfdB2*	y=−4.1217×+23.306	0.98	y = −5.7735× + 30.019	0.98	0.98 ± 0.02
*’c gfdB3*	y=−4.0371×+22.878	0.98	y = −5.5362× + 30.126	0.99	0.97 ± 0.13

^a^In these qPCR analyses, *Aspwe1_39921* encoding the ortholog of the *A. nidulans* γ-glutamylcysteine synthetase (*A. nidulans* locus ID: AN3150) gene was used as a single copy reference gene. The number of incorporated *A. nidulans gfdB* (locus ID: AN6792) gene(s) per genome was determined by the following equation: *gfdB* per genome = (total copies of *gfdB*)/(total copies of *Aspwe1_39921*). ^b^The equation C_T_ =m(log quantity) + b was constructed by plotting the standard curve of log quantity versus its corresponding C_T_ value, where, y is the C_T_value, m is the slope, x is the log(quantity), and b is the intercept

**Fig. 1:**
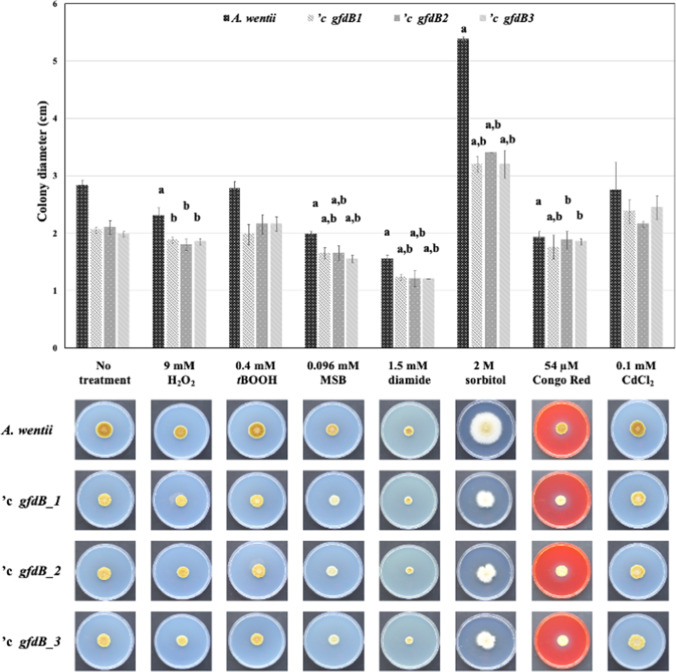
Stress sensitivity phenotypes of the *A. wentii* wild-type and the *gfdB*-complemented *'c gfdB1*, *'c gfdB2*, and *'c gfdB3* strains under various stress conditions, after 10 d incubation at 25 °C on NMM stress agar plates. In each experiment, letters “a” and “b” indicate significant differences between the growths of stress treated and untreated cultures and significant interactions between the effects of genetic manipulations and stress exposures, respectively

**Fig. 2:**
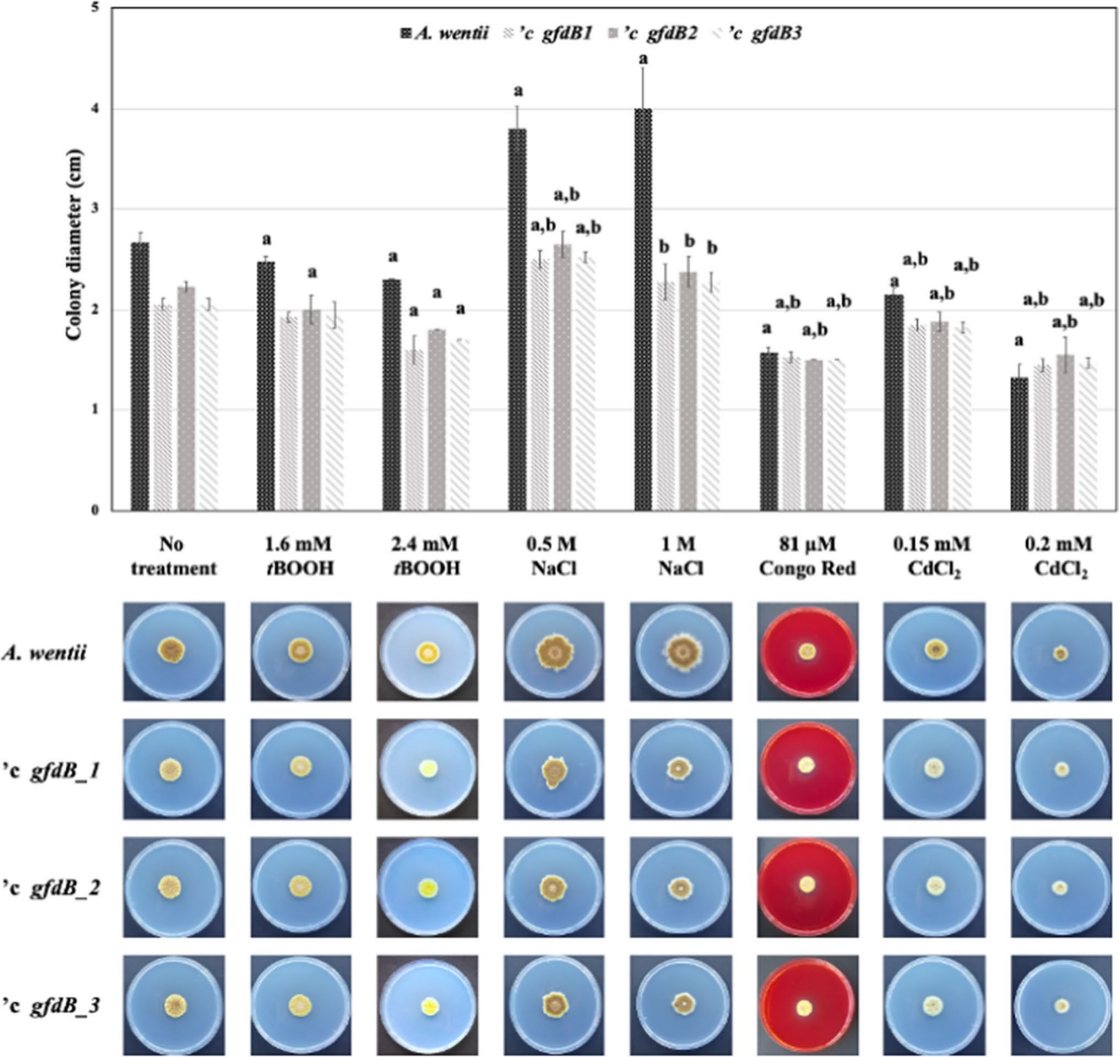
Varying stress tolerance of the *A. wentii* wild-type and the *gfdB* complemented *'c gfdB1*, *'c gfdB2*, and *'c gfdB3* strains (10 d incubation, 25 °C, NMM stress agar plates). Letters “a” and “b” stand for significant differences between the growths of stress treated and untreated cultures and significant interactions between the effects of genetic manipulations and stress exposures, respectively

**Fig. 3:**
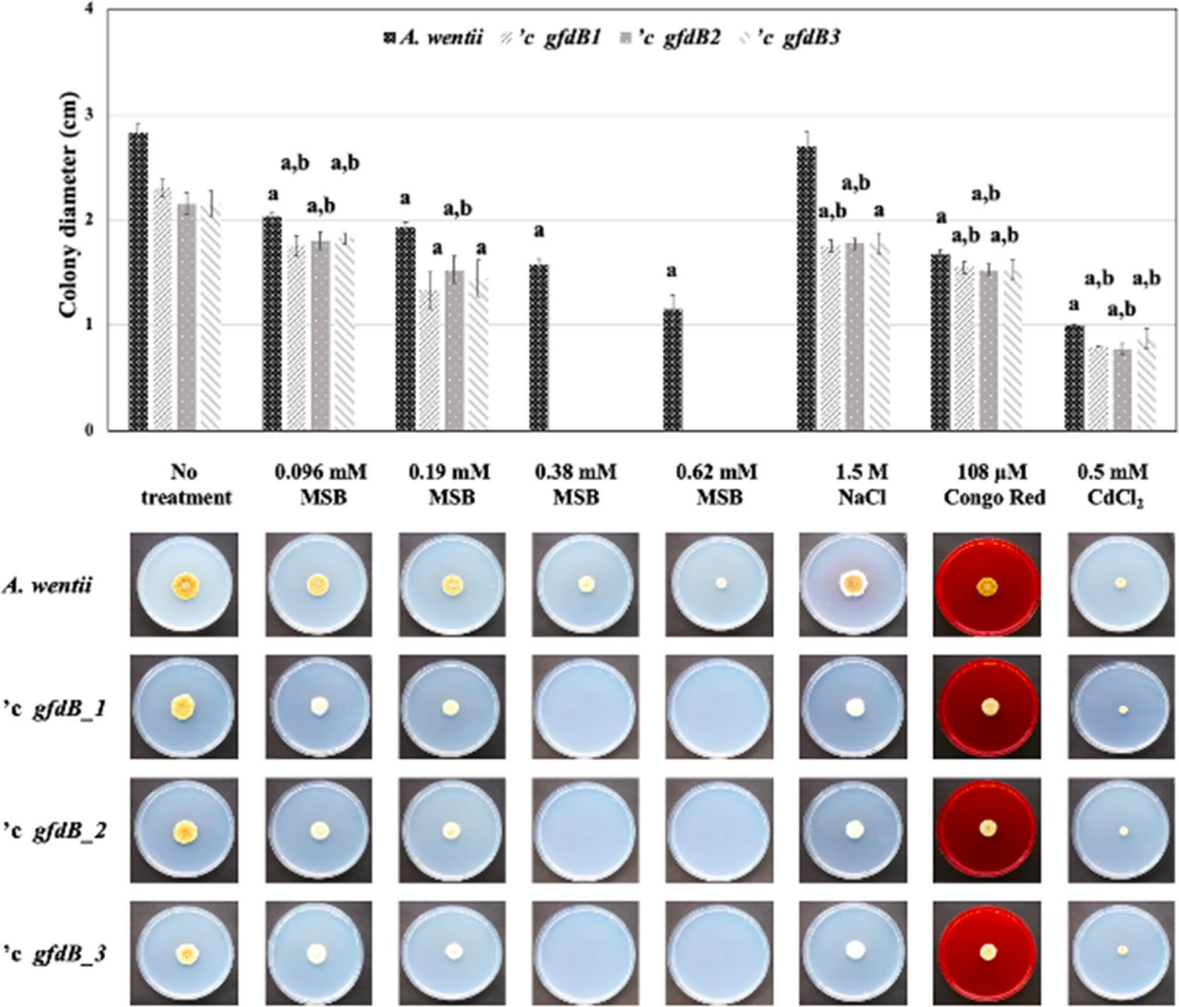
Stress phenotypes of the *A. wentii gfdB* complemented *'c gfdB1*, *'c gfdB2*, and *'c gfdB3* strains (10 d incubation, 25 °C, NMM stress agar plates). “a” significant differences between the growths of stress treated and untreated cultures. “b” significant interactions between the effects of genetic manipulations and stress exposures

**Fig. 4:**
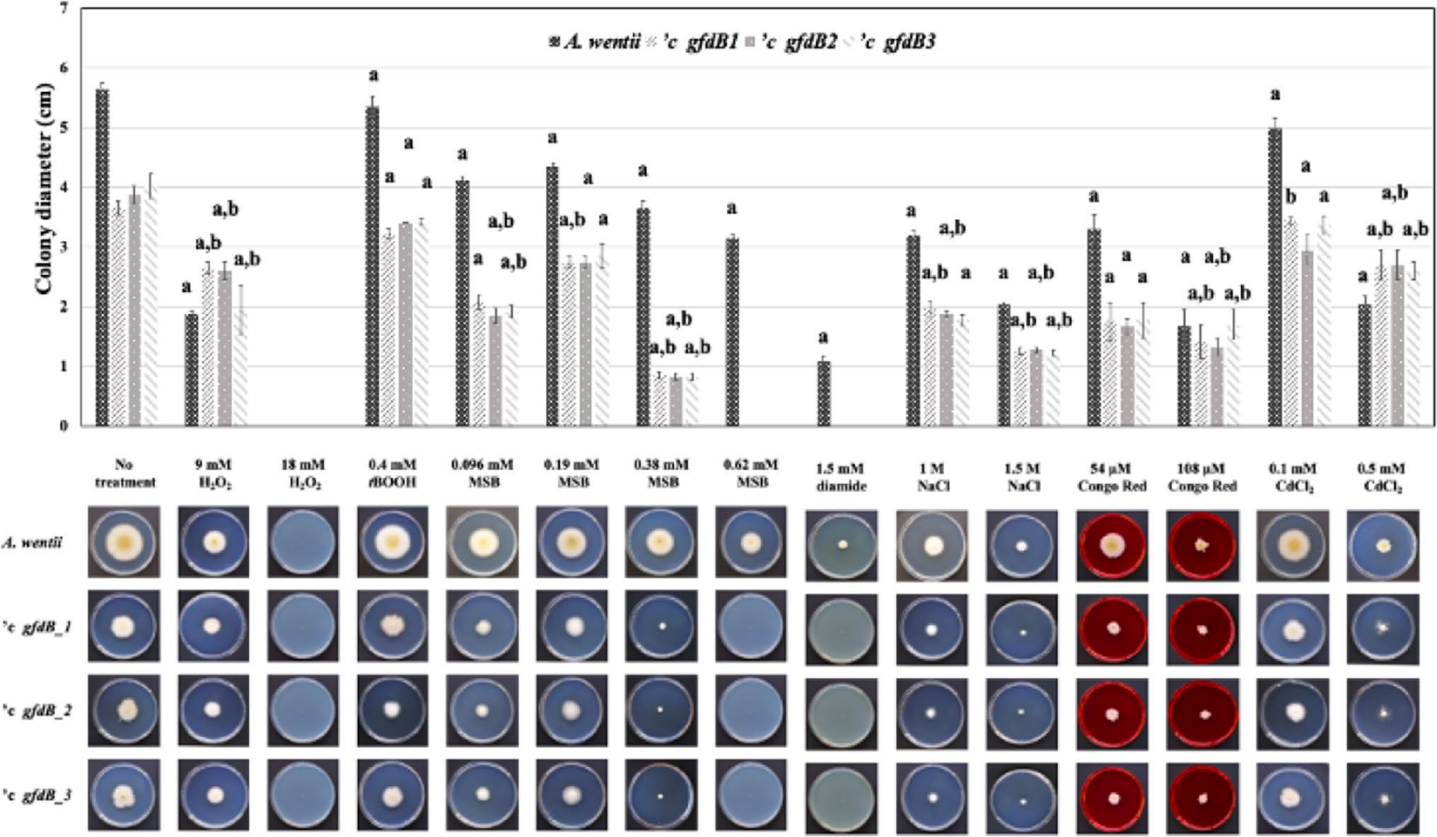
Versatile stress sensitivity phenotypes recorded in *A. wentii gfdB* complemented *'c gfdB1*, *'c gfdB2*, and *'c gfdB3* strains, grown on NMM stress agar plates (10 d, 25 °C, in the presence of 2 M sorbitol). “a” significant differences between the growths of stress treated and untreated cultures. “b” significant interactions between the effects of genetic manipulations and stress exposures

The osmophily and the stress sensitivity phenotypes of the *A. wentii 'c gfdB1*, *'c gfdB2*, and *'c gfdB3* strains (independent transformants) were compared to those of the *A. wentii* wild-type strain*.* Remarkably, the supplementation of *A. wentii* with *A. nidulans gfdB* partially complemented the osmophily of wild-type *A. wentii* exposed to 2 M sorbitol or to 0.5 M or 1 M NaCl (Figs. 1 and 2), meanwhile exposure to a higher (1.5 M) NaCl concentration did not result in any osmophily in the wild-type strain and was even inhibitory for the *’c gfdB* strains (Fig. 3). A slow growth phenotype was observed for the *gfdB* supplemented *A. wentii* mutants in the absence of sorbitol with appr. 16–30% decreases in the colony diameters (Figs. 1, 2, and 3). In the presence of 2M sorbitol, unstressed colony diameters were appr. 28–35% smaller for the *A. wentii 'c gfdB1*, *'c gfdB2*, and *'c gfdB3* mutant strains than those recorded in the case of the *A. wentii* wild type strain (Fig. 4). Some sporadic minor stress sensitivity phenotypes were observed in oxidative (H_2_O_2_, *t*BOOH, MSB, diamide), cell wall integrity (Congo Red), and heavy metal (Cd^2+^) exposed *A. wentii* cultures which were typically enhanced in the *’c gfdB* strains with the exception of high (0.2 mM) concentration CdCl_2_ treatments when the supplementation of *A. nidulans gfdB* mildly mitigated the observed heavy metal stress sensitivity (Figs. 1 and 2).

Combined osmolyte treatments (2 M sorbitol with 1 M or 1.5 M NaCl) were disadvantageous for *A. wentii* and the inhibitory power of the osmolyte mixtures was further enhanced in the *’c gfdB* strains (Fig. 4). In some cases, the addition of 2 M sorbitol to culture media increased the severity of environmental stress, e.g., the *’c gfdB* strains did not even grow out in the presence 1.5 mM diamide (Fig. 4), which stress treatment gave us only a minor phenotype on NMM stress agar without any sorbitol supplementation (Fig. 1). In contrast, 2 M sorbitol helped the *’c gfdB* strains to grow out when exposed to 0.38 mM MSB (Fig. 4), while no outgrowth of the *gfdB* supplemented *A. wentii* strains was recorded in the absence of the osmolyte (Fig. 3). Interestingly, the addition of 2 M sorbitol to NMM agar also slightly increased the oxidative stress tolerance of the *A. wentii ’c gfdB* strains in the presence of 9 mM H_2_O_2_ (Fig. 4), which was not observed in NMM stress agar experiments in the absence of sorbitol (Fig. 1).

Interactions between stress exposures and *A. nidulans gfdB* supplementations were also investigated by two-way ANOVA followed by Tukey’s post hoc test. As shown in Figs. 1, 2, 3, and 4, stress exposure - *gfdB* interactions were rather sporadic but some clear-cut interactions (marked by letter “b” in the upper parts of the figures) were recorded. Interestingly, no interaction between 2.4 mM *t*BOOH treatment and *gfdB* supplementation was observed in *A. wentii* (Fig. 2) although *A. nidulans gfdB* fully restored the growth of *A. glaucus* exposed to 0.4 mM *t*BOOH on stress agar plates supplemented with 2 M sorbitol (Király et al. 2020b). The addition of 2 M sorbitol did not influence these interactions considerably in *A. wentii* but the 54 μM Congo Red treatment – *gfdB* supplementation interaction (Fig. 1) was lost in the presence of 2 M sorbitol (Fig. 4).

### Stress tolerance-based positioning of *A. glaucus* and *A. wentii* among aspergilli

**Fig. 5:**
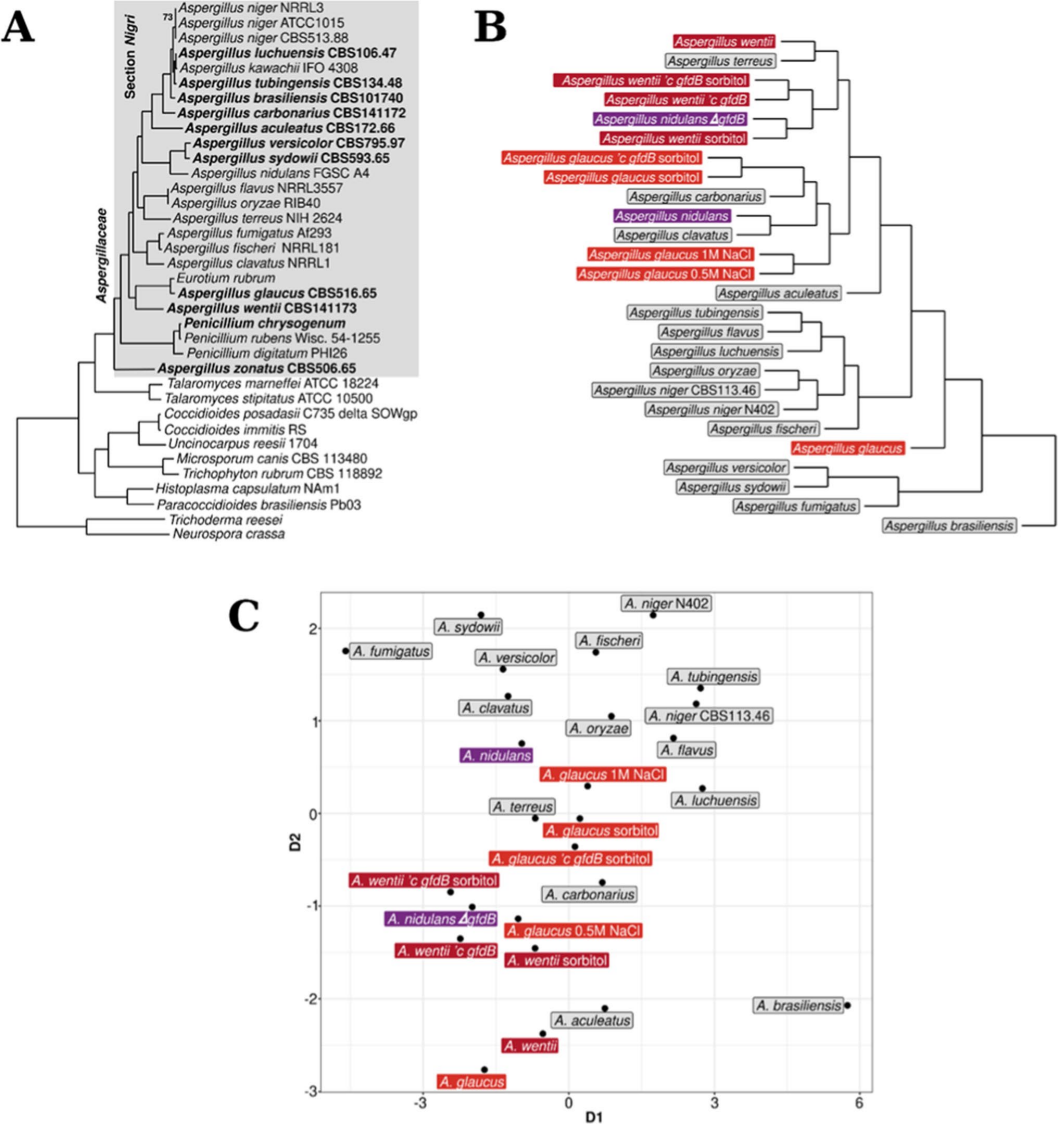
Comparison of the phylogenetic positions and the stress tolerance of the tested *Aspergillus* strains. Part A, maximum likelihood phylogeny of the *Aspergillus* spp. as reproduced from the previous publication of de Vries et al. (2017) with modifications. Note that *A. niger* ATCC 1015 is identical to CBS 113.46. Part B, cluster analysis dendrogram constructed on the stress tolerance data gained for *A. glaucus*, *A. nidulans*, and *A. wentti* strains in current and previous (Király et al. 2020a,b) studies or reposited in the Fungal Stress Database (all other strains; Orosz et al. 2018; http://www.fung-stress.org/). Part C, multidimensional scale plot presentation of the stress tolerance variability of the *Aspergillus* species tested (de Vries et al. 2017; Emri et al. 2018)

The evolutionary distances of 17 selected *Aspergillus* species (*A. niger* was represented by two strains) based on stress sensitivity patterns publicly available in FSD (Orosz et al. 2018) were visualized by the generation of multidimensional scale plots using the MIC_50_ values (H_2_O_2_, MSB, CdCl_2_) and colony diameters measuered at selected concentrations (sorbitol, NaCl, Congo Red) as a result of current and previous stress tolerance studies (Emri et al. 2018). In addition, cluster analysis was also performed to construct dendrograms (Fig. 5; 5 and 10 d incubations, 25 °C; Emri et al. 2018).

In this study, cluster analysis and multidimensional scaling were performed on an extended stress database to track changes in the stress tolerance-based positioning of the two osmophilic species *A. glaucus* and *A. wentii*, which were elicited by osmolytes and *gfdB* supplementation. To reach this aim, stress sensitivity data gained in *A. glaucus* and *A. wentii* NMM stress agar cultures supplemented with 0.5 and 1 M NaCl (*A. glaucus*), with 2 M sorbitol (*A. glaucus* and *A. wentii* wild-type strains, *A. glaucus* and *A. wentii ’c gfdB* strains) (de Vries et al. 2017; Emri et al. 2018; Orosz et al. 2018; Király et al. 2020b), as well as those recorded with the *A. nidulans ΔgfdB* gene deletion mutant (Király et al. 2020a) were also taken into consideration in the construction of both the dendogram (Fig. 5B) and the multidimensional scale plot (Fig. 5C).

Neither the construction of a dendrogram via cluster analysis (Fig. 5B) nor multidimensional scale plot presentation of the distances between the environmental stress tolerance of the tested *Aspergillus* spp. (Fig. 5C) separated remarkably well the osmolyte exposed and *gfdB* supplemented *A. glaucus* and *A. wentii* strains from the wild-type *A. wentii*, wild-type *A. nidulans*, and the *A. nidulans ΔgfdB* strains. Nevertheless, *A. wentii* and *A. nidulans ΔgfdB* both lacking *gfdB* genes were relatively closer to each other than to wild-type *A. nidulans* (Fig. 5). No similar tendencies were observed for *A. glaucus*, which is a strictly xerophilic/osmophilic fungus that can hardly grow without the supplementation of any osmolyte (Supplemental Fig. S2; de Vries et al. 2017; Orosz et al. 2018; Király et al. 2020b).

As shown in Fig. 5B and C, all *A. wentii* wild-type and *’c gfdB* strains grown on either NMM or 2 M sorbitol supplemented NMM agars remained in the proximity of *A. terreus* (Fig. 5B; cluster analysis dendrogram) or close to *A. aculeatus* (Fig. 5C; multidimensional scale plot presentation). Similarly, all osmolyte (NaCl, sorbitol) supplemented *A. glaucus* strains remained close to *A. carbonarius* and *A. clavatus* (Fig. 5B; cluster analysis dendrogram) or in the proximity of *A. carbonarius* and *A. terreus* (Fig. 5C; multidimensional scale plot presentation).

### Stress tolerance-based positioning of the tested *A. glaucus*, *A. wentii*, and *A. nidulans* strains

**Fig. 6:**
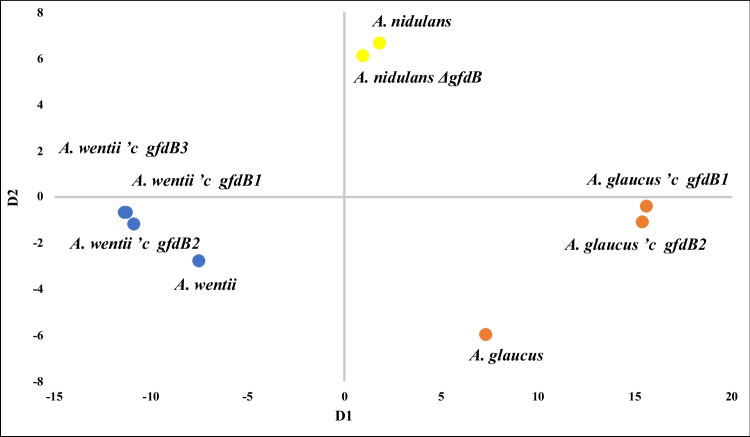
Multidimensional scale plot presentation of the stress tolerance variability of the *Aspergillus* wild-type (*A. nidulans*, *A. glaucus*, *A. wentii*) and mutant (*A. nidulans ΔgfdB*; *A. glaucus 'c gfdB1* and *'c gfdB2*; *A. wentii 'c gfdB1*, *'c gfdB2*, and *'c gfdB3*) strains tested. Stress sensitivity assays were carried out on NMM stress agar plates (10 d incubation, 25 °C) and culture media prepared for the *A. glaucus* strains always contained 2 M sorbitol

The stress tolerances of *A. glaucus* and *A. wentii* wild-type and *’c gfdB* mutant strains were also compared to those of the *A. nidulans* wild-type and *ΔgfdB* mutant strains *via* the generation of multidimensional scale plots. In this set of experiments, colony diameters measured on NMM agar plates supplemented with selected stress initiating agents (54 μM Congo Red, 2 M sorbitol, 0.096 mM MSB, 0.4 mM *t*BOOH, 9 mM H_2_O_2_, 1 M NaCl, and 0.5 mM CdCl_2_) were taken into consideration (Fig. 6). Unlike the 17 *Aspergillus* species-based cluster analysis dendrogram and multidimensional scale plot presented in Fig. 5, this approach clearly indicated that the supplementation of *A. wentii* and *A. glaucus* with the *A. nidulans gfdB* gene increased the distance between these species (Fig. 6). This result is in line with the observation that the insertion of *gfdB* massively increased the oxidative stress tolerance of *A. glaucus* without influencing its osmophily (Király et al. 2020b), while the insertion of the same gene into the *A. wentii* genome reduced osmophily without giving a clear, unidirectional change in its oxidative stress tolerance (Figs. 1, 2, 3, and 4). Interestingly, the same method did not show profound differences in the stress tolerance of the *A. nidulans* wild-type and *ΔgfdB* strains, and both strains remained separated, far apart from the *A. glaucus* and *A. wentii* strains tested (Fig. 6).

## Discussion

Fungi are exposed to various environmental stresses, such as heat shock, oxidative, and osmotic stress, and glycerol has an important role in overcoming various stress conditions and microenvironments. Glycerol 3-phosphate dehydrogenase (G3PDH) can catalyze the reversible redox conversion of dihydroxyacetone phosphate to glycerol 3-phosphate, which is then dephosphorylated into glycerol. However, current knowledge on the functions of *G3PDH* genes in aspergilli is limited (Zhang et al. 2018). Nevertheless, the ancient G3PDH-encoding *gfd* gene was duplicated before the diversification of the ascomycetous fungal species belonging to the genus *Aspergillus* (Miskei et al. 2009; Balázs et al. 2010; de Vries et al. 2017), and the potentially harmful disturbance in *gfd* gene dosage seems to be successfully resolved by various subfunctionalization and neofunctionalization events (Wapinski et al. 2007; Ames et al. 2010; Levasseur and Pontarotti 2011; Emri et al. 2018).

Previous studies clearly indicated that *A. nidulans gfdA* and *gfdB* found their important physiological functions in the maintenance of cellular growth and cell wall integrity (*gfdA*; Fillinger et al. 2001) and in oxidative stress defence (*gfdB*; against H_2_O_2_, *t*BOOH, and diamide; Király et al. 2020a). It is important to note that the physiological functions of *A. nidulans gfdA* and *gfdB* did not separate hermetically because the *ΔgfdB* strain also showed minor reduction in growth and cell wall integrity (Congo Red) phenotypes (Király et al. 2020a).

The loss of one of the *gfd* paralogs is a relatively rare event in aspergilli but two xerophilic/osmophilic species, *A. glaucus* and *A. wentii*, evolutionarily lost their *gfdB* orthologous gene (de Vries et al. 2017). Therefore, it was reasonable to assume a causal connection between this gene loss event and the appearance of osmophily (de Vries et al. 2017; Orosz et al. 2018). This hypothesis was further strengthened by the upregulation of *gfdB* (but not *gfdA*) under 0.6 M NaCl exposure in *A. nidulans* (Balázs et al. 2010).

In a previous study by Király et al. (2020b), we managed to supplement *A. glaucus* with *A. nidulans gfdB* resulting in *’c gfdB* strains with considerably increased *t*BOOH and, to a lesser extent, increased H_2_O_2_, MSB, Congo Red, and CdCl_2_ tolerance. Since *A. glaucus* is a promising enzyme (Tao et al. 2010, 2011; Abrashev et al. 2016; Li et al. 2018; Takenaka et al. 2019; Chen et al. 2020) and polyketide (Cai et al. 2009, 2014; Sun et al. 2009; Wu et al. 2017) producer and bioremediation (Gajendiran and Abraham 2017; Wei and Zhang 2018; Zhou et al. 2021) fungus, and a satisfactory stress tolerance is highly recquired for any industrial fungal strains (Bai et al. 2003; Li et al. 2011; Teixeira et al. 2011; Hagiwara et al. 2016; Deparis et al. 2017; Steensels et al. 2019; Brandt et al. 2021; Yaakoub et al. 2022), these observations raised the question if the contribution of *A. nidulans gfdB* to oxidative stress tolerance could be exploited in other *Aspergillus* spp. as well.

After supplementation of *A. wentii* with *A. nidulans gfdB* gene, only minor changes in the stress tolerance were observed in the *A. wentii ’c gfdB* strains in comparison to the *A. wentii* wild-type strain, including slightly increased CdCl_2_ (Figs. 1 and 2) as well as oxidative (MSB, H_2_O_2_; only in the presence of 2 M sorbitol; Figs. 1, 2, 3, and 4) stress tolerance. Therefore, we reached the conclusion that these sporadic and hardly significant improvements in environmental stress tolerance would be difficult to take advantage of under industrial conditions (Sinha and Chakrabarty 1978; Chander et al. 1980; Gross et al. 1984; Shoaib et al. 2018; Lago et al. 2021).

Stress tolerance-based positioning of *A. glaucus* and *A. wentii* among the aspergilli was achieved using cluster analysis dendrogram and multidimensional scale plot presentation approaches as shown in Fig. 5B and C, respectively. It is noteworthy that the tested *A. wentii* and all osmolyte supplemented *A. glaucus* wild-type and *’c gfdB* strains took their positions near some industrially and/or agriculturally important *Aspergillus* spp. including *A. aculeatus* (a promising hydrolase producing fungus; Mhuantong et al. 2020; Wang et al. 2021), *A. carbonarius* (a major ochratoxin A producer in grapes; Mondani et al. 2020), *A. clavatus* (a rich source of secondary metabolites; Zutz et al. 2013), or in the proximity of *A terreus* (producing lovastatin, itaconic acid and hydrolyses; Ryngajłło et al. 2021). Therefore, further studies should aim at shedding light onto any possible link between the environmental stress tolerance of these fungi and the presence of a *gfdB* ortholog in their genomes (de Vries et al. 2017; Emri et al. 2018; Orosz et al. 2018).

When stress tolerance-based positioning of the tested *A. glaucus*, *A. wentii*, and *A. nidulans* strains was carried out using data obtained after a careful selection of stress initiating agents, these species sperated well and the supplementation of *A. wentti* and *A. glaucus* with *A. nidulans gfdB* increased the distance between these *Aspergillus* species, meanwhile their positions relative to the *A. nidulans* wild-type and *ΔgfdB* strains remained essentially unaltered (Fig. 6).

These observations on the stress tolerance-based positions of the tested *Aspergillus* spp. and their mutants warn us that any alterations in the stress response systems of fungi may trigger rather complex, often unpredictable physiological changes. Furthermore, we can determine the size and direction of the phenotypic changes only based on the results of a large number of carefully executed experiments, after carrying out properly chosen mathematical analyses.

Industrial fungi are confronted with a wide spectrum of environmental stress factors including oxidative stress (Bai et al. 2003; Li et al. 2011; Teixeira et al. 2011; Hagiwara et al. 2016; Deparis et al. 2017; Steensels et al. 2019; Brandt et al. 2021; Yaakoub et al. 2022). Moreover, some aspergilli (*A. glaucus*, *A. wentii*, *A. versicolor*, *A. sydowii*, and *A. oryzae*) show osmophily under various culture conditions (de Vries et al. 2017; Orosz et al. 2018). A deeper understanding of the molecular background of osmophily may pave the way for the development of new, highly stress tolerant industrial strains (Király et al. 2020a,b). Future screening studies in submerged *Aspergillus* cultures on stress-elicited changes in the concentrations of intracellular compatible solutes, e.g., glycerol, mannitol, erythritol, and arabitol (Sánchez-Fresneda et al. 2013; de Lima Alves et al. 2015; Király et al. 2020a) will likely provide ways to increase sugar alcohol yields in industrial fermentation processes — an important field mostly dedicated to osmotolerant/osmophilic yeasts thus far (Moon et al. 2010; Yang et al. 2021; Erian and Sauer 2022; Yaakoub et al. 2022).

The evolution of the stress response system of fungi is remarkably fast especially at the level of transcriptional regulation (Nikolaou et al. 2009; Zhang et al. 2016; Emri et al. 2018). We can also assume that the *A. nidulans gfdB* inserted with its own promoter into the genomes of *A. glaucus* and *A. wentii* (Fig. 5A) interacts with different transcription factors with altered stress responsiveness and modified promoter preferences (Wohlbach et al. 2009), contributing to the observed phenotypic differences of the *A. glaucus* and *A. wentii c’ gfdB* strains (Figs. 1, 2, 3, and 4; Király et al. 2020b). Future studies should therefore focus on the development of suitable tools for the constitutive expression of *A. nidulans gfdB* gene using high-expressed promoter and terminator sequences of host fungi, like *A. wentii*.

## Supplementary information

ESM 1 (PDF 697 kb)

## Data Availability

The datasets generated during and/or analyzed during the current study are available from the corresponding author on reasonable request.
