# Supplementary material for: Species-specific effects of the introduction of Aspergillus nidulans gfdB in osmophilic aspergilli
Source: Appl Microbiol Biotechnol. 2023 Feb 22;107(7-8):2423–36. doi: 10.1007/s00253-023-12384-9 (PMC10033484; doi:10.1007/s00253-023-12384-9)
Supplement: Supplementary file 1 — (PDF 697 kb) [file 253_2023_12384_MOESM1_ESM.pdf]

Applied Microbiology and Biotechnology

Supplemental Material

Species-specific effects of the introduction of *Aspergillus nidulans gfdB* in osmophilic aspergilli

Veronika Bodnár[1,2], Anita Király[1], Erzsébet Orosz[1], Márton Miskei[3], Tamás Emri[1,3], Zsolt Karányi[4], Éva Leiter[1,3], Ronald P. de Vries[5] and István Pócsi[1,3,*]

Affiliations

[1] Department of Molecular Biotechnology and Microbiology, Institute of Biotechnology, Faculty of Science and Technology, University of Debrecen, Debrecen, Hungary

[2] Doctoral School of Nutrition and Food Sciences, University of Debrecen, Debrecen, Hungary

[3] ELRN-UD Fungal Stress Biology Research Group, Debrecen, Hungary

[4] Department of Medicine, Faculty of Medicine, University of Debrecen, Debrecen, Hungary

[5] Fungal Physiology, Westerdijk Fungal Biodiversity Institute & Fungal Molecular Physiology, Utrecht University, Utrecht, the Netherlands

Corresponding author

*I. Pócsi, e-mail: pocsi.istvan@science.unideb.hu, telephone: +36-52-512900 ext. 62337, ORCID ID: 0000-0003-2692-6453

Supplemental Tables

Supplemental Table S1. Oligonucleotides used in this study

**Name**	**Sequence (5’-3’)**	**Purpose**
	**Primers used to generate complemented strains**	
*AN6792 Xba*I FW	ggtctagaGTATCGAGGCTGGATTGGTGTAAAGTGATT	forward primer of the *AngfdB* gene
*AN6792 Hin*dIII Rev	ttaagcttCTTCATGATCCCTGTGGCGCATGTAGACGC	reverse primer of the *AngfdB* gene
	**Primers used in copy number determinations**	
AN6792 FW	CCAGTATCCTCGTCTTCAAC	forward primer of the *AngfdB* gene
AN6792 Rev	TCGTCTCGCATAGTCTTTC	reverse primer of the *AngfdB* gene
Aspwe1_39921 FW	ATACACTGCCCCTCACATTCTG	forward primer of the *γ-glutamylcysteine synthetase* gene
Aspwe1_39921 Rev	CCTCATAGCGGTCGGTCATAG	reverse primer of the *γ-glutamylcysteine synthetase* gene
	**Primers used in qRT-PCR experiments**	
gfdB63 FW	GCCAGTATCCTCGTCTTCAACC	forward primer of the *AngfdB* gene
gfdB63 Rev	TGTCGTCTCGCATAGTCTTTCG	reverse primer of the *AngfdB* gene
Aspwe1_38228 FW	CCGAGGACCGTTACAAGG	forward primer of the *Afutef1* gene
Aspwe1_38228 Rev	GGACAGGAGGCTCAATGG	reverse primer of the *Afutef1* gene

Supplemental Table S2. Standardized MIC_{50} values and colony diameters from stress tolerance studies used to generate MDS plot and dendrogram (Figs. 5B and 5C).

**Species/strain**	**Control 25 °C 5 d**	**Control 25 °C 10 d**	**H_2O_2 MIC_{50} 25 °C 5 d**	**H_2O_2 MIC_{50} 25 °C 10 d**
A. aculeatus	0.57483	-0.35711	-0.28896	-0.36956
A. brasiliensis	1.45251	1.59707	0.27150	-0.02246
A. carbonarius	-0.05208	0.13144	-0.13497	0.08470
A. clavatus	0.07330	-0.16169	-0.55822	-0.43560
A. fischeri	1.82866	1.54821	0.56577	1.05176
A. flavus	1.70328	0.66884	1.40186	0.95484
A. fumigatus	0.70022	0.08258	-1.58867	-1.76057
A. glaucus	-2.43436	-1.96930	0.00000	0.00000
A. glaucus 0.5M NaCl	-1.55668	-0.79680	-0.47139	-0.74389
A. glaucus 1M NaCl	-0.92976	-0.65023	0.18849	0.13769
A.glaucus 2M sorbitol	0.95098	1.59707	-0.60160	-0.81297
A. luchuensis	0.44945	0.37571	1.18672	0.72385
A. nidulans	0.82560	0.71769	-0.88248	-0.38116
A. niger CBS113.46	0.07330	0.61998	2.00088	2.04630
A. niger N402	-0.42823	0.03373	2.02720	1.54625
A. oryzae	-0.05208	-0.35711	1.36418	2.14805
A. sydowii	0.07330	-0.11283	-0.39837	-0.77948
A. terreus	-0.55361	-0.35711	-0.02903	0.30016
A. tubingensis	0.82560	1.25509	0.84320	0.63419
A. versicolor	-0.92976	-1.23649	0.37734	0.53610
A. wentii	-0.80438	-1.43190	-0.76373	-0.81297
A. wentii 2M sorbitol	0.07330	0.03373	-1.05210	-0.58579
A.glaucus 'c gfdB 2M sorbitol	0.24048	1.40979	-0.56248	-0.75425
A. wentii 'c gfdB	-1.18053	-1.67618	-0.78691	-0.88180
A. wentii 'c gfdB 2M sorbitol	-0.80438	-0.80901	-0.87817	-0.74815
A. nidulans ΔgfdB	-0.11895	-0.15518	-1.23006	-1.07522

**Species/strain**	**MSB MIC_{50} 25 °C 5 d**	**MSB MIC_{50} 25 °C 10 d**	**$CdCl_2$ MIC_{50} 25 °C 5 d**	**$CdCl_2$ MIC_{50} 25 °C 10 d**
A. aculeatus	1.93028	1.99370	-0.78227	-0.61941
A. brasiliensis	3.75272	3.21851	-0.75253	-0.54488
A. carbonarius	1.29475	0.59901	-0.78186	-0.65828
A. clavatus	-0.58103	-0.60240	0.68161	0.44017
A. fischeri	-0.28323	-0.52272	-0.18177	0.42629
A. flavus	-0.06444	-0.10424	-0.36308	-0.21927
A. fumigatus	-0.97083	-1.10745	3.15936	1.16726
A. glaucus	-0.95214	-1.10991	-0.67096	-0.64326
A. glaucus 0.5M NaCl	-0.76861	-0.84409	-0.58567	-0.59251
A. glaucus 1M NaCl	-0.83655	-0.96999	-0.66986	-0.73077
A.glaucus 2M sorbitol	-0.10917	-0.52615	-0.57540	-0.75162
A. luchuensis	-0.14945	-0.40449	-0.50086	-0.18669
A. nidulans	-0.53166	-0.40571	0.05845	-0.03279
A. niger CBS113.46	-0.40384	-0.02470	0.41463	0.01475
A. niger N402	-0.31770	-0.12147	0.31248	0.33320
A. oryzae	-0.06980	-0.35498	0.00251	-0.12480
A. sydowii	-0.48634	-0.58674	1.44766	3.07993
A. terreus	-0.10955	0.61454	1.14969	-0.03211
A. tubingensis	-0.15617	-0.27423	-0.43427	-0.56815
A. versicolor	0.12947	1.76121	2.21413	2.86547
A. wentii	0.21927	0.26028	-0.69976	-0.62383
A. wentii 2M sorbitol	0.66719	0.68548	-0.37101	-0.56061
A.glaucus 'c gfdB 2M sorbitol	-0.00969	-0.29927	-0.53651	-0.75529
A. wentii 'c gfdB	-0.29021	-0.30785	-0.66582	-0.35688
A. wentii 'c gfdB 2M sorbitol	-0.66627	-0.38590	-0.07516	0.38403
A. nidulans ΔgfdB	-0.23701	-0.18043	-0.79373	-0.70994

**Species/strain**	**Sorbitol 25 °C 5 d**	**Sorbitol 25 °C 10 d**	**NaCl 25 °C 5 d**	**NaCl 25 °C 10 d**	**Congo Red 25 °C 5 d**	**Congo Red 25 °C 10 d**
A. aculeatus	0.99918	-0.38027	-2.12715	-0.74119	0.00000	0.18548
A. brasiliensis	1.82526	2.08874	-0.05230	3.03284	1.25416	1.64584
A. carbonarius	-0.15733	-0.83962	-0.05230	-0.40422	0.47672	0.38462
A. clavatus	-1.31384	-0.78220	-0.05230	-0.06725	-0.45620	0.78290
A. fischeri	-0.81820	0.13650	-0.67476	0.06753	-0.92266	0.11910
A. flavus	0.50353	0.88295	-0.67476	-0.06725	1.25416	0.25186
A. fumigatus	-1.14863	-1.29897	-2.12715	-1.34773	-2.01107	-0.94298
A. glaucus	1.52787	2.06003	0.36267	0.40450	-2.01107	-2.00506
A. glaucus 0.5M NaCl	0.50353	-0.15059	0.00000	0.00000	-0.30071	0.31824
A. glaucus 1M NaCl	-0.65298	-0.03576	0.00000	0.00000	1.25416	1.44670
A.glaucus 2M sorbitol	0.00000	0.00000	0.10331	-1.02761	0.32124	0.31824
A. luchuensis	1.16440	1.34230	2.02255	1.41540	0.94318	0.84928
A. nidulans	-0.68602	-1.20710	0.17593	-0.26269	-0.45620	0.05272
A. niger CBS113.46	1.49483	0.82553	0.36267	0.13493	1.09867	1.04842
A. niger N402	-1.31384	-0.03576	0.98512	1.34801	1.09867	0.78290
A. oryzae	0.00789	-0.38027	-0.46727	-0.67379	0.78770	0.18548
A. sydowii	-0.81820	-0.60995	1.40009	0.87625	-0.76717	-0.54470
A. terreus	-0.15733	0.02166	0.36267	0.80886	-0.61169	-1.27488
A. tubingensis	-0.65298	0.94036	0.57015	0.47189	1.56513	2.04412
A. versicolor	-0.48776	-0.60995	0.15518	-0.13465	-0.30071	-0.61108
A. wentii	1.32961	0.29440	1.40009	0.20232	-0.45620	-0.81022
A. wentii 2M sorbitol	0.00000	0.00000	-0.41540	-0.47161	-0.30071	-1.14212
A.glaucus 'c gfdB 2M sorbitol	0.00000	0.00000	0.15518	-0.85912	0.41453	0.05936
A. wentii 'c gfdB	-0.36385	-0.95446	-0.05230	-1.09500	-0.14522	-0.97617
A. wentii 'c gfdB 2M sorbitol	0.00000	0.00000	-1.66031	-1.31403	-0.65056	-1.05915
A. nidulans ΔgfdB	-0.78515	-1.30758	0.30042	-0.29639	-1.07815	-1.10893

Note: The table contains standardized values. These values are derived from MIC_{50} and other stress tolerance measurements. A negative number indicates below-average, while a positive number indicates higher-than-average stress tolerance.

Supplemental Figures

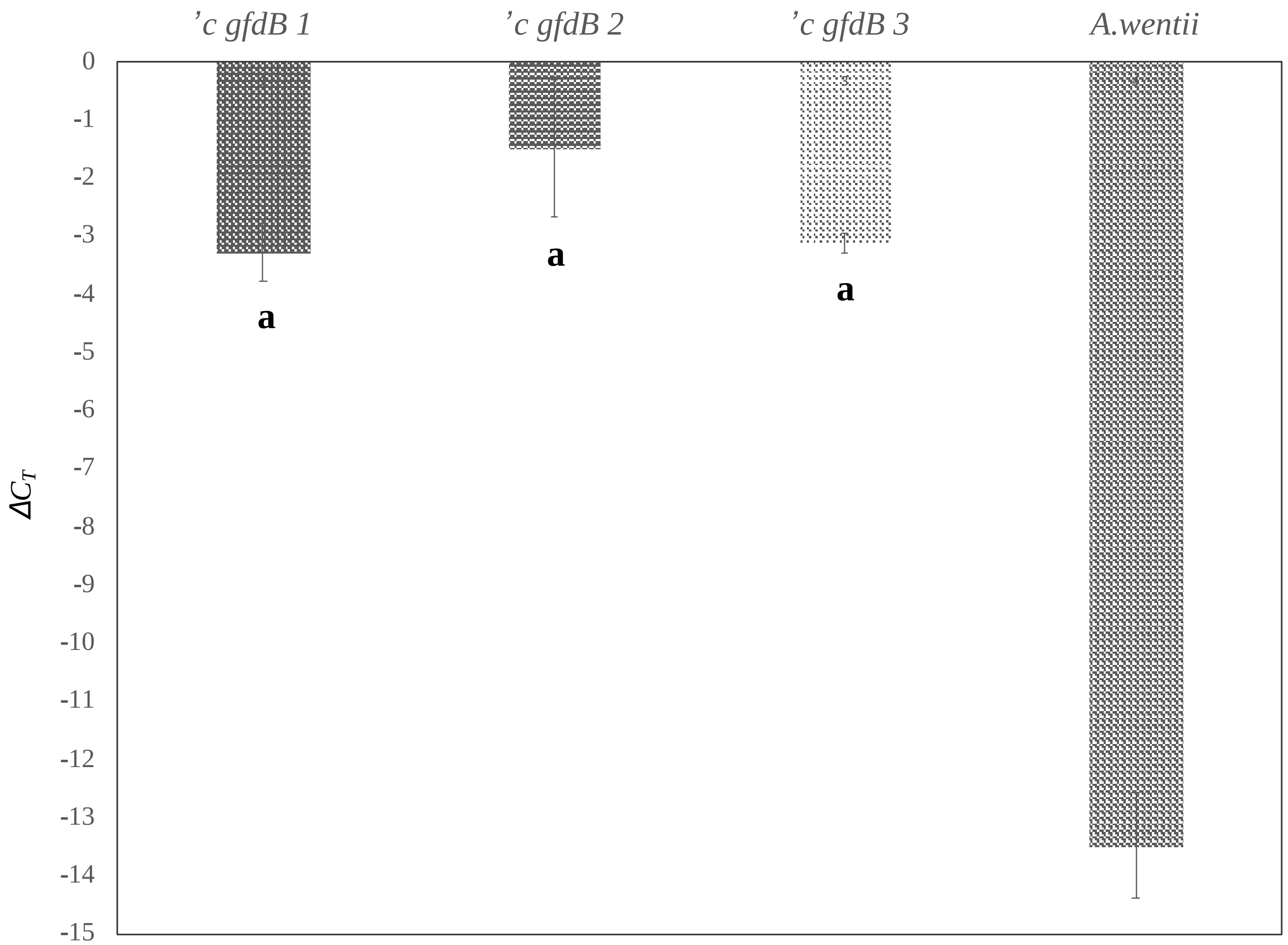

Supplemental Figure S1. Relative transcription levels of the *gfdB* gene determined in the *A. wentii 'c gfdB1*, *'c gfdB2* and *'c gfdB3* mutants and, in the wild type strain (CBS141173). Mycelia were harvested from cultures incubated in nitrate minimal medium at 220 rpm and 25°C for 3 days. Relative transcription levels were quantified with the ΔΔCP method, where ΔC_T = C_T reference gene − C_T *gfdB*, and C_T stands for the qRT-PCR cycle numbers corresponding to the crossing points. The *Aspwe1_38228* gene (ortholog of the *A. fumigatus tef1*) was used as reference gene. Means calculated from three independent experiments are presented and bars show SD values.

[a] – Significant difference between the mutant and the wild type strain ($p < 0.05$; Student *t*-test). The relative transcription values of the mutants did not differ significantly from one and other ($p < 0.05$; Student *t*-test).

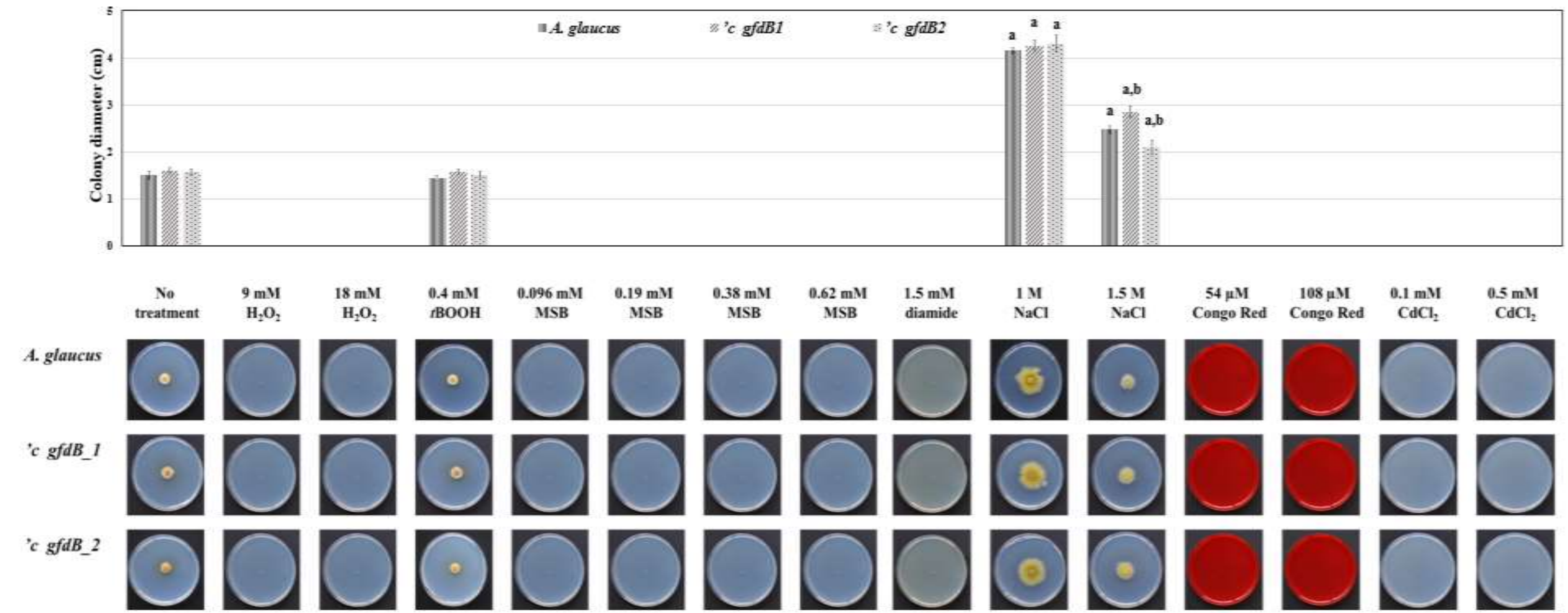

Supplemental Figure S2. Stress phenotypes of the *A. glaucus gfdB* complemented *'c gfdB1* and *'c gfdB2* strains (10 d incubation, 25 °C, NMM stress agar plates). “a”: significant differences between the growths of stress treated and untreated cultures. “b”: significant interactions between the effects of genetic manipulations and stress exposures.

Note: In a separate set of experiments, we supplemented the already available stress sensitivity data for *A. glaucus* and the appropriate *A. glaucus 'c gfdB* strains. As expected, *A. glaucus* hardly grew on NMM stress agars without the supplementation of any osmolytes like NaCl or sorbitol. Unexpectedly, some minor growths were recorded in the presence of *t*BOOH.
